# Quality Assurance in Laparoscopic Gynecological Training: A Narrative Review of Standards with a Multicenter Expert Perspective on Pathways Toward Effective Education and Care

**DOI:** 10.3390/clinpract16090165

**Published:** 2026-09-05

**Authors:** Vlad Iustin Tica, Liliana Steriu, Dragos Brezeanu, Andrei A. Tica, Ana-Maria Brezeanu, Diana Badiu, Roxana Penciu, Silvia Onuc, Irina Tica, Maya Sophie de Wilde

**Affiliations:** 1 Department of Obstetrics and Gynecology, Doctoral School, University Emergency County Hospital of Constanța, “Ovidius” University of Constanța, Bd. Tomis 140, 900591 Constanta, Romania; vtica@eeirh.org (V.I.T.); lilianasteriu@yahoo.com (L.S.); anmariataras@gmail.com (A.-M.B.); dianabadiu@yahoo.com (D.B.); roxanapenciu@yahoo.com (R.P.); silviaizvoranu@yahoo.com (S.O.); 2 Academy of Romanian Scientists, 3 Ilfov, 050044 Bucharest, Romania; 3 Department of Pharmacology, University of Medicine and Pharmacy of Craiova, Craiova County Emergency Hospital, Str. Tabaci 1, 200534 Craiova, Romania; 4 Department of Internal Medicine, Doctoral School, University Emergency County Hospital of Constanța, “Ovidius” University of Constanța, Bd. Tomis 140, 900591 Constanta, Romania; irinatica73@yahoo.com; 5 Department of Obstetrics and Gynecology, Carl von Ossietzky University, Pius-Hospital, 26121 Oldenburg, Germany; sophie.dewilde@pius-hospital.de

**Keywords:** quality assurance, medical education, laparoscopic surgery, gynecological endoscopy, postgraduate training, surgical competency, curriculum standards

## Abstract

**Background:** Quality assurance (QA) is a cornerstone of safe and effective postgraduate training in gynecological laparoscopic surgery, where clinical outcomes depend on procedural complexity, equipment sophistication, and the coordinated competence of an entire surgical team. This article is a narrative review of the conceptual, professional, and regulatory foundations of QA in healthcare education, complemented by a structured synthesis of the collective teaching and clinical experience of the author group. It is not an empirical study, and the sections that follow present a conceptual mapping supported by expert perspective rather than original outcome data. **Methods:** Sources were identified through targeted searches of PubMed/MEDLINE and Google Scholar, supplemented by hand-searching of policy and curriculum documents issued by professional bodies and by backward citation tracking, without date restriction. Approximately 480 records were screened at title and abstract level, 112 were assessed as full text, and 41 sources were retained against predefined inclusion and exclusion criteria, with five further sources added during peer review. In a second and separate step, the ten co-authors, who hold teaching and supervisory roles in gynecological laparoscopy across academic centers in Romania and Germany, each completed the same six-item written prompt on training provision, trainer development, trainee assessment, and institutional QA practice; responses were coded thematically by two authors independently and consolidated into explicit points of convergence and divergence. **Results:** We describe how QA principles, originally articulated in general quality-management and health-promotion literature, have been adapted by professional and regulatory bodies, including the European Board and College of Obstetrics and Gynecology (EBCOG), European Society for Gynecological Endoscopy (ESGE), American Society for Gastrointestinal Endoscopy (ASGE), and the UK General Medical Council, into concrete standards for training providers, trainers, and trainees. We outline the structures used to monitor and audit training quality, including Kirkpatrick’s four-level evaluation model and periodic institutional review cycles, alongside documentation and certification pathways such as the Gynecological Endoscopic Surgical Education and Assessment (GESEA) program, with governance and financing as cross-cutting determinants of sustainability. The author synthesis converged on a consistent gap between documented standards and their routine enforcement and diverged on whether external review and formal certification should become mandatory. **Conclusions:** The conceptual architecture of QA in gynecological laparoscopy is coherent and broadly fit for purpose, but we identified no study linking the implementation of a specific QA standard to measurable gains in trainee competency or patient outcomes in this field. Proposals for harmonized, outcome-oriented, and where appropriate, mandatory external review should therefore be read as reasoned positions awaiting prospective evaluation rather than as evidence-based recommendations; generating that evidence, with the trainee’s own perspective collected rather than inferred, is the principal task for future research.

## 1. Introduction

Quality is not an abstraction in clinical training: it is the standard against which patient safety, professional competence, and the credibility of an entire specialty are ultimately measured. In gynecological endoscopy, quality depends on the coordinated contribution of many actors across the whole patient journey, from admission to discharge, and on the willingness of every team member, surgical and non-surgical, to sustain that standard: cleaning staff who keep the preoperative environment fit for purpose, operating-room teams who keep equipment functional, and endoscopic teams who ensure that surgeons have reliable instruments at hand [1,2]. Because laparoscopic equipment is sophisticated and outcomes are sensitive to operator skill, training is not peripheral to quality in this field; it is one of its principal levers. In recognition of this, professional bodies such as the European Board and College of Obstetrics and Gynecology (EBCOG) have taken on an explicit QA role, including through the Standing Committee on Training Recognition, which can evaluate laparoscopic training in countries that lack a national evaluation system of their own [3].

Quality Assurance (QA) follows naturally from this premise. Training is an essential component of any defined standard of care, and QA is the operational mechanism by which educational and clinical systems identify needs, design interventions, and verify that a genuinely high standard of care is being delivered [3]. The integration of QA into both healthcare delivery and medical education illustrates this interdependence well: pursued together, the two processes have a greater impact on outcomes than pursued in isolation [4]. Beyond the clinical benefit, the visible use of QA motivates the people involved and allows training institutions to progress toward their full potential, to the benefit of trainers, trainees, and patients alike [3,4,5].

A demarcation should be stated at the outset rather than left to the limitations. Several of the foundational QA concepts used in this article—the definition of a quality standard, the audit cycle, and the distinction between structure, process, and outcome—were developed in general quality-management, health-promotion, and emergency-care literature, not in surgical education [6,7,8]. We treat them as transferable in principle, but the transfer is an argument rather than a demonstration, and wherever a concept is imported from outside surgical training we identify it in the text as conceptual. Standards developed specifically for gynecological laparoscopy—EBCOG’s European Training Requirements and the EBCOG-PACT curriculum, the ESGE/GESEA certification pathway, and ASGE’s endoscopy quality indicators—are identified as such where they appear [3,9,10]. The reader should weigh the two categories differently, and the limits of concept transfer are revisited in Section 8.

As a primary objective, this article summarizes the conceptual basis of QA as applied to healthcare and medical education. Secondary objectives are to translate these principles into concrete standards for the training provider, the trainer, and the trainee in gynecological laparoscopy; to describe how training quality is monitored, evaluated, audited, and documented; and to identify the gaps that should guide future research and policy in this field. Two points about what follows should be clear in advance. First, Section 3, Section 4, Section 5, Section 6 and Section 7 present a narrative synthesis of published standards and frameworks together with the author group’s expert perspective; they do not report empirical findings, and the section headings should be read in that light. Second, this article is distinct in scope from our earlier work on standards of care in gynecologic laparoscopic surgery, which argued for standardization at the level of surgical practice and its consequences for training. Here the unit of analysis is the training system itself: we set out the QA standards that apply to providers, trainers, processes, and trainees; we add a structured multicenter synthesis of how those standards are actually applied in two European countries, including the points on which the authors disagree (Section 4.5 and Table 1); and we identify the absence of outcome-linked evidence as the field’s principal research gap rather than as a passing caveat.

## 2. Methods

### 2.1. Design and Terminology

This article is reported as a narrative review with an accompanying structured synthesis of expert opinion. It is not a systematic review, and we make no claim to exhaustive coverage of the literature. Throughout, standard refers to a threshold of expected quality agreed between providers and beneficiaries; indicator refers to a measurable proxy used to judge whether that threshold has been met; and quality assurance refers to the systematic process that links the two. Section 4.5 and Table 1 report the author synthesis; all other sections report published sources. Where both appear in the same paragraph, each sentence states which of the two it rests on.

### 2.2. Literature Component

Sources were identified through targeted searches of PubMed/MEDLINE and Google Scholar, run between March and June 2025, using combinations of the terms “quality assurance,” “quality indicators,” “medical education,” “postgraduate training,” “laparoscopic training,” “gynecological endoscopy,” “competency-based medical education,” “simulation,” and “surgical curriculum.” These were supplemented by hand-searching of institutional and policy documents central to the field—the EBCOG European Training Requirements and the EBCOG-PACT curriculum, ESGE/GESEA curriculum materials, ASGE quality-indicator publications, and UK educational governance documents (General Medical Council, Royal College of Radiologists, and regional commissioning guidance)—and by backward citation tracking of the retrieved articles. No date restriction was applied, because several foundational definitions of QA in health education predate the laparoscopic-training literature itself.

Approximately 480 records were identified and screened at title and abstract level by two authors (D.B. (Dragos Brezeanu) and A.-M.B.), of which 112 were assessed as full text; 41 sources were retained from this process. A further five sources were added during peer review at the reviewers’ suggestion, and one superseded policy document was replaced by its current version, giving 46 references in total; sources published after the search window entered the article by this route rather than through the original searches. Inclusion criteria were: (i) direct relevance to healthcare or educational QA, postgraduate surgical education, competency-based assessment, or gynecological laparoscopic training; (ii) status as a peer-reviewed article, professional guideline, accreditation standard, or educational framework issued by a recognized body; and (iii) publication in English. Exclusion criteria were: (i) single case reports and conference abstracts without an available full text; (ii) commentary without an identifiable conceptual or empirical contribution; (iii) sources addressing surgical technique alone, with no educational or QA dimension; and (iv) superseded versions of a policy document, of which only the current version was retained. Where two sources supported the same point equally well, the one closer in scope to gynecological laparoscopy was cited. Because selection was purposive rather than exhaustive, no formal risk-of-bias appraisal and no PRISMA-type selection process were undertaken, and selection bias cannot be excluded; this is revisited in Section 8.

### 2.3. Expert Reflection Component

The second component of this article is a structured synthesis of the experience of the ten authors, all of whom hold teaching, supervisory, or clinical leadership roles in gynecological laparoscopy in academic training centers in Romania (Constanța, Craiova) and Germany (Oldenburg). Reflections were collected by written response to a standardized prompt, circulated by electronic mail to all ten authors in May 2025; all ten returned a completed response. The prompt was identical for every author and comprised six predefined open questions:

(1) In your center, which QA standards for laparoscopic training are formally documented, and which are applied in practice without being documented?
(2) How, and by whom, is a trainee’s readiness to perform a step, or a whole procedure, without direct supervision decided?
(3) What simulation resources are available to trainees, how is access organized, and what limits their use?
(4) How is formative feedback structured in daily practice, and is any explicit model (for example, Pendleton’s rules) used?
(5) How is the trainee logbook or portfolio verified, at what intervals, and what follows if requirements are not met?
(6) What role do external review and formal certification pathways (for example, GESEA) play in your center, and what obstacles do you encounter?

Responses were free text of approximately 200–800 words per question. Two authors (D.B. (Dragos Brezeanu) and A.A.T.) independently read all ten responses and coded them inductively, generating an initial code list that was grouped into five themes: documentation versus enforcement; entrustment and supervision; simulation access; feedback culture; and external review and certification. The two coders compared their coding, discussed discrepancies, and agreed a final framework; a third author (V.I.T.) arbitrated where agreement was not reached. Within each theme, statements were classified as convergent (reported in comparable form by authors from both countries), partially convergent (reported by several authors but with material differences in form or degree), or divergent (reported differently or reported by authors from one country only). The output of this process is presented in Section 4.5 and summarized in Table 1.

This is a transparent but not a validated consensus procedure. It is not a Delphi study: there was no iterative re-circulation of aggregated responses, no anonymized re-rating, and no quantitative measure of agreement such as a kappa statistic or a predefined consensus threshold. Retrospective conversion to a Delphi process would require fresh data collection and was not undertaken. The synthesis should therefore be read as structured expert reflection with a documented method, and its limits are set out in Section 8.

## 3. Conceptual Framework: Quality and Quality Assurance in Healthcare

### 3.1. Defining Quality and Quality Assurance

Quality assurance is commonly defined as all the planned and systematic actions necessary to provide adequate confidence that a product or service will satisfy given requirements for quality, a definition rooted in the ISO 9000 family of quality-management standards [6]. QA is both a process and a tool: it allows continuous monitoring, maintenance, and improvement of an activity, and it seeks better results even where quality is already comparatively high.

Within a defined structure or procedure, QA ensures that quality standards are implemented with reliability, validity, and reproducibility, and it supports reporting in both quantitative terms, where suitable indicators exist, and qualitative ones [7]. QA addresses not only structures and processes but also subjective outcomes, such as patient satisfaction, alongside the objective outcomes defined within the process itself [8]. In healthcare, QA is instrumental in sustaining the community’s trust that the highest available standard of professional care is being provided and received [8]; it does so by emphasizing proper clinical protocols, adequately trained personnel, and a properly functioning working environment that extends beyond infrastructure or instrumentation alone. The concept has gained sufficient recognition in medicine to be the subject of dedicated manuals covering standards and criteria, and it is generally considered a more complete process descriptor than the clinical audit alone, a distinction equally valid in medical research [11,12].

In endoscopy specifically, the American Society for Gastrointestinal Endoscopy (ASGE) has adopted programs that develop quality indicators measurable at both the patient and procedural level [9]. In gynecological laparoscopy, a competency-based training curriculum with explicit indicators, a recognized training-recognition process, and structured faculty-development programs together provide the backbone of QA [3].

### 3.2. Quality Standards and Their Monitoring

Quality standards define the threshold of quality expected for a process or its results, agreed by both providers and beneficiaries [12]. In healthcare, as in other fields, two complementary sets of generic standards are useful: the first covers strategic planning, program management, and monitoring/evaluation, reflecting core management activities such as the identification of needs and objectives, process design and assessment, and outcome analysis; the second covers education and training, resources and information, and consultancy, and forms the prerequisite core that this review addresses [9,13].

Monitoring of quality standards is essential to their implementation and should occur at every level, performed by both implementers and beneficiaries. The process itself comprises the development of criteria, an evaluation schedule, and data generation and analysis, criteria that are, in effect, descriptive and measurable standards, used to judge the degree to which the primary quality standards are being met [12].

Practical implementation of QA requires, among other elements, a clear definition of quality, the development of standards and outcome indicators, measurement systems, recruitment and training of QA personnel, and clear allocation of tasks and coordination across the people, processes, and organizations involved [14]. In gynecological laparoscopy, this translates into well-balanced training programs that cover the full scope of obstetrics and gynecology, an appropriate trainee-to-trainer ratio, and adherence to the working-hours limits set by the European Working Time Directive [3].

The implementation strategy itself proceeds through several concomitant or sequential sub-processes: correct design with quality standards embedded from the outset, clear motivation and tactical goals, selection of assessors based on prior performance, and frequently partnership arrangements that bring in complementary expertise, followed by data entry and analysis [1,7,15]. A well-known example of this strategy in action is the Global Rating Scale (GRS), developed by the UK’s National Health Service in 2004 as the first scoring system to assess QA in endoscopy by measuring parameters such as communication, informed consent, procedure and pathology reporting, and facility accessibility; its introduction was associated with reduced waiting times and adverse events and increased patient satisfaction, and the model was subsequently adapted and adopted elsewhere in Europe and in Canada [9,16].

Although quality assurance is often described through multiple overlapping concepts, its practical implementation can be understood through several core domains that collectively support educational and clinical excellence. In gynecological laparoscopic training, these domains encompass structural resources, training processes, outcome assessment, monitoring mechanisms, and continuous improvement strategies. The main dimensions of quality assurance and their relevance to laparoscopic training are summarized in Table 2.

## 4. Quality Assurance Applied to Training in Gynecological Laparoscopy

Because training is itself a process subject to QA, the standards described above translate into specific expectations for the training provider, the trainer, the training process itself, and the trainee.

### 4.1. Standards for the Training Provider

All stages of training are subject to QA in pursuit of two linked goals: the safety and fitness for practice of trainees, and the assurance owed to providers, participants, and beneficiaries alike that the quality of a medical act does not depend on who happens to perform it [4]. Embedding QA principles into the design of training, as a personal objective of trainers and trainees alike, helps make quality control “a legitimate part of the job” rather than an external imposition [17].

Quality standards for the training provider are the responsibility of the training institution, together with the steps required to meet them. The institution’s educational capacity, its resources, staff, trainee population, and training management should be openly advertised to trainers, trainees, and the public, and assessed through partnership with similar institutions. A clearly and openly stated institutional commitment to QA, visible at every level, should underpin this capacity. A central instrument for QA in laparoscopy is the institutional development and acquisition of laparoscopic simulators, which improve the skills and attitudes of trainees who will, in turn, become the next generation of trainers [3,18].

Staff recruitment, induction, development, support, supervision, and monitoring form a further set of standards intended to ensure that training institutions are properly resourced. Personnel competence and accreditation should be pursued through continuous professional development, both as subject experts and as educators, and institutional responsibility extends to working conditions and contractual matters such as trainers’ leave, retirement, illness, pregnancy, and the handling of disputes and complaints. Effective staff monitoring relies on observation, self-assessment, and a systematic analysis of trainees’ feedback.

The trainee occupies the central position in the educational program and should be supported through well-designed advertising, information before, during, and after training, and counseling. Equality and diversity of opportunity, selection, and treatment, including compliance with relevant anti-discrimination legislation, together with health and safety conditions must be actively implemented and monitored [19]. Learners’ needs should be identified early, and the procedures to address them should follow; the learning environment—intellectual, physical, and procedural—should be conducive to learning and adapted to trainees with disabilities [19]. The systematic analysis of trainers’ feedback, already mentioned, remains of central importance throughout.

At the institutional level, QA indicators should track staff growth, the organization of periodic continuing-education courses, and the introduction of laparoscopy into the residency curriculum; staff appreciation, promotion, structured feedback on job satisfaction, and team-building activities that strengthen communication between providers and staff are all part of this picture [9,20]. Further managerial standards address data collection, processing, archiving, and periodic reporting; participation monitoring; compliance with relevant legislation on education, health and safety, working time, data protection, confidentiality, and human rights; staff liability policy; and premises insurance. Educational staff, equipment, and facilities should be suitable for, and proportionate to, the number of trainees enrolled, and effective links should be maintained between theoretical subjects and practical training [19].

### 4.2. Standards for the Trainer

Standards for the trainer concern experience, qualification and expertise, both in the clinical subject area and in education itself, accreditation, continuous professional development with appropriate record-keeping, and enthusiasm for teaching [19]. Punctuality, ongoing attention to health and safety issues, and prompt communication of any change in the training program are further, more operational, quality criteria.

Endoscopic trainers in gynecology should be demonstrably competent to teach the procedures they supervise, and a training checklist should be used to assess that teaching ability [9,18]. To sustain quality in laparoscopic training, trainers themselves should participate in continuing courses designed to develop their teaching competency, not only their procedural skill [3].

Continuing professional development is not an obligation that lapses once a surgeon becomes experienced, and treating it as a trainee concern misses one of its principal functions. Techniques, energy platforms, and instrument systems change over the course of a career, and a trainer who has not kept pace transmits, in good faith, a version of practice that is no longer current; the duty to update is therefore also a duty toward the next generation of surgeons [21,22]. Structured CPD for senior surgeons, periodic re-accreditation and update courses, participation in proctored sessions when a new procedure is introduced, and documented attendance at faculty-development activities addressing teaching as well as technique should accordingly be treated as a quality standard for the trainer in its own right, recorded and reviewed as such, rather than left to individual initiative [3,5]. The same logic applies to the transfer of new knowledge downward: a department in which senior surgeons attend update courses but have no forum in which to pass on what they learned has met the letter of the CPD requirement and not its purpose.

### 4.3. Standards for the Training Process

Generic process standards, common to virtually all training programs, include safety considerations for patients, trainees, and trainers; observance of institutional policies and procedures; a clear, mutually understood, and adhered-to statement of learning outcomes; timely and suitable information provided before, during, and after training; and regular quality management, monitoring, and evaluation [14,19]. Training itself should be grounded in the principles of adult learning—motivation (needs assessment and planning toward desired outcomes), reinforcement through practical experience, retention through reflection and evaluation, and transference into clinical practice—and should integrate the cognitive, skills, and attitudinal domains of learning, with the trainee’s overall experience treated as an outcome in its own right.

Contemporary postgraduate surgical education has progressively shifted from time-based training models toward competency-based and outcome-based educational frameworks [22]. In competency-based medical education (CBME), progression is determined by the achievement of predefined competencies rather than by duration of training alone [23]. Within gynecological laparoscopy, this approach aligns closely with quality assurance principles because competency acquisition, procedural performance, and patient safety outcomes become measurable indicators of educational quality [24]. Entrustable Professional Activities (EPAs), milestone-based assessment systems, and structured competency portfolios may provide objective mechanisms for evaluating trainee progression and supporting continuous quality improvement within training programs [25,26].

Training organizations should use structured methods to monitor trainees’ learning and progression; participation in recurring international events, such as those organized annually by the European Society for Human Reproduction and Embryology (ESHRE), is one practical example. Evidence-based criteria should underpin the choice of learning resources and the standards used to assess training quality, so that the training itself models the evidence-based practice it is meant to teach [27].

Pre-training standards cover the provision of accurate, accessible, impartial, and comprehensible information about training opportunities, a non-discriminative and candidate-adaptable enrollment process, transparent information on costs (including ancillary costs and any financial assistance available), and, where feasible, information on post-training career pathways [14,19]. From induction onward, training standards address health and safety information, the support and complaint procedures available to trainees, and a training plan that includes assessment of trainers, trainees, and the program itself, ensuring that trainees can adapt the pace of training to their individual level, use their time efficiently, and access the facilities, materials, and guidance they need [19].

Centering the process on the trainee means actively encouraging learners to take responsibility for co-designing their own training path, including extracurricular learning, and for their own progress, built on a constructive relationship with trainers and the institution and on effective two-way feedback [14,27]. Trainers, for their part, should cultivate empathy, unconditional positive regard, confidence, and respect toward trainees, and trainees should be actively encouraged to propose improvements to the training program, its materials, and its schedule; laparoscopic simulators should be used routinely as part of this iterative improvement [4,5,9,28,29].

Managing the training process day to day is assessed against a further set of practice- and result-oriented criteria: how well the process adapts to individual trainees in pace and plan, the strength of the links between theory and practice, the frequency of formative checks and intermediate summaries, the accuracy of participation and behavior monitoring, and the degree to which the learning climate remains stimulating and appropriately challenging [14,19]. Educational tools deserve specific mention here: early training typically relies on mechanical simulators, while advanced training may progress to live-animal models; available evidence suggests that skill acquisition and overall QA both improve when live-animal training is incorporated at the appropriate stage [28].

A related process standard, often underestimated, concerns familiarity with the specific devices in use. Laparoscopic performance depends not only on generic psychomotor skill but on knowing the behavior of the energy device, stapler, insufflator, or optical system present in a given operating room: its setup, its failure modes, and the tactile and visual cues it generates. Recent comparative work in gynecological laparoscopy reports that operative outcomes for a given pneumoperitoneum and entry technique differ appreciably between experienced surgeons and trainees, which indicates that competence with a particular device or manoeuvre is acquired rather than assumed [30]. Training programs should therefore treat device-specific orientation, ideally on a bench model or simulator before the first supervised clinical use, as a defined step in the training pathway rather than something acquired incidentally at the operating table, and should repeat it whenever a new platform is introduced into the department [3]. This applies to established surgeons as much as to trainees, and it is a point at which the trainer standards of Section 4.2 and the process standards described here meet.

Interrelations between specialties and institutions are themselves a quality dimension. Links to other specialties and to personal-development opportunities support the horizontal transferability of skills across subspecialties, professional groups, and geographic settings—an especially relevant consideration in an interconnected field such as obstetrics and gynecology, and in the context of increasing cross-border mobility among doctors [14,27]. A genuine partnership between trainers, trainees, academic and health institutions, and the public is the foundation on which the acceptability of any QA system ultimately rests.

Evaluation deserves separate emphasis: it is not only a tool used to assess training quality but an integral part of training itself, and accordingly a distinct group of standards addresses supervision, appraisal, and assessment. Appraisal is informal and supportive, oriented toward personal planning and improvement; assessment is more formal, more standardized, and tests defined aspects of process and outcome against predetermined criteria. Assessment occurs at several stages: initial (establishing the trainee’s starting level), formative (ongoing), and summative (final)—and should be fair, equally applied, dependable, and methodologically valid, ensuring concordance between the initial goals and expectations of trainers and trainees and the results achieved.

Gynecological laparoscopic training itself typically proceeds through a defined sequence: basic endoscopic skill training, exposure to teamwork and operating-room practice, advanced skill training, and a stepwise progression from simple to complex procedures under close supervision; entry into unsupervised operating-room practice should follow demonstrated knowledge and skill, certified accordingly [3]. The equipment used should span pelvic trainers, models for laparoscopic psychomotor and suturing skills (separately and combined), and simulation models for specific procedures [3].

Throughout this evaluation process, feedback on trainees’ progress should be positive, timely, non-judgmental, focused on the action rather than the person, and informed by the trainee’s own experience. Pendleton’s rules offer a practical structure for this: the trainee first describes their own achievements, difficulties, and coping strategies, after which the trainer provides positive feedback and constructive, specific suggestions for improvement. Post-training priorities include the timely award of certificates and achievements and continued involvement in institutional or collaborative projects relevant to the trainee’s development.

### 4.4. Standards for the Trainee

Standards for the trainee largely mirror, and are represented by, the training-process standards already described. Simulation-based and standardized laparoscopic training curricula should be embedded in residency programs to improve the skills of future laparoscopic surgeons [18], and diagnostic laparoscopy itself should be a formal part of residency training in every country, so that fewer patients are subjected to exploratory laparotomy by the cohort of trainees now entering practice [20].

At the postgraduate level, more formal standards apply. In the UK, the General Medical Council (GMC) sets mandatory standards for the organizations responsible for postgraduate education, grouped into themes that cover the learning environment and culture, educational governance and leadership, supporting learners, and supporting educators [31]. At the European level, the first training curriculum in obstetrics and gynecology was adopted by EBCOG in 1998 and has been periodically updated since; it defines the discipline as encompassing the supervision of pregnancy and delivery and the diagnosis and treatment of disorders of the female reproductive organs, including the pelvic organs and breast [3].

The successful implementation of quality assurance requires coordinated participation from all stakeholders involved in the educational process. Responsibilities are distributed across institutions, trainers, trainees, accrediting organizations, and healthcare systems, each contributing specific elements that support training quality and patient safety. The principal responsibilities and associated quality standards are presented in Table 3.

**Figure 1:**
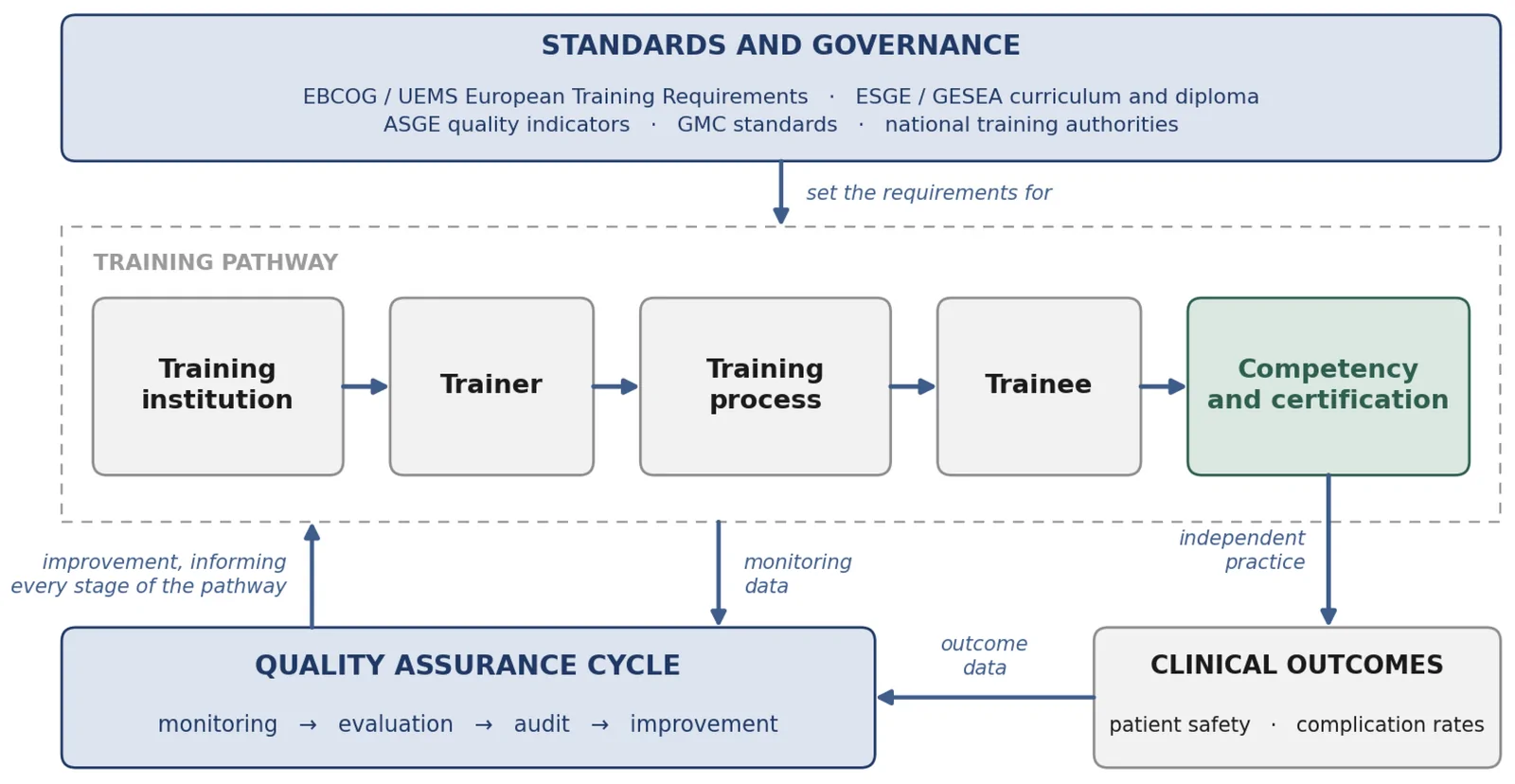
Simplified framework of quality assurance (QA) in gynecological laparoscopic training. Standards and governance—European Training Requirements, the ESGE/GESEA curriculum and diploma, ASGE quality indicators, GMC standards, and national training authorities—set the requirements for a training pathway running from the training institution through the trainer, the training process, and the trainee to competency and certification, and from there to independent practice and clinical outcomes. A single QA cycle of monitoring, evaluation, audit, and improvement closes the loop from those outcomes back to every stage of the pathway.

Taken together, these standards and responsibilities describe a continuous cycle rather than a linear sequence: the educational pathway from the training institution to clinical outcomes is sustained by an ongoing quality assurance loop of monitoring, evaluation, audit, and improvement, as shown in Figure 1.

### 4.5. Synthesis of Author Experience: A Multicenter Perspective

The standards described in Section 4.1, Section 4.2, Section 4.3 and Section 4.4 are drawn from the published literature; how they are applied from day to day varies across institutions in ways that published frameworks rarely capture. This section reports the synthesis of the ten authors’ written reflections, collected and coded as described in Section 2.3. Everything that follows in this section is expert opinion and is explicitly distinguished from the literature reviewed above. The five subsections correspond to the coding framework, and Table 1 sets out, theme by theme, where the authors converged and where they did not.

Documentation versus enforcement. The most consistent finding across all ten responses was a gap between what is formally documented and what is routinely enforced. Every center represented has written requirements covering trainee-to-trainer ratios, logbook content, and the expectation of structured feedback; none has a written escalation procedure specifying what happens when those requirements are not met, so the consequence in practice depends on the individual supervisor. The clearest concrete difference between countries concerns the timing of verification. At the German center (Pius-Hospital, Oldenburg), logbook review is tied to the annual structured appraisal interview, which creates a recurring and actionable checkpoint at which a shortfall can still be corrected. At the Romanian centers (Constanța and Craiova), verification is predominantly retrospective and concentrated at the end of the training period, by which point a documented shortfall in operative numbers cannot realistically be remedied within the residency. The authors were unanimous that the interval of verification, rather than the content of the requirement, is the variable that most affects whether the requirement functions. This observation reinforces, from practice, the argument made in Section 4.1 that monitoring must occur at every level and on a defined schedule.

Entrustment and supervision. Across all centers, the decision to allow a trainee to perform a step, or a whole procedure, without direct supervision is made by the individual supervisor based on observed performance, and not against a written, program-level entrustment scale. No center represented here uses a formal Entrustable Professional Activity framework for laparoscopy, although the EBCOG-PACT curriculum provides for entrustment-based assessment [10]. A practical consequence, reported independently by several authors, is that trainees who rotate between centers experience a noticeable difference in the pace at which they are entrusted, and interpret this as a property of the supervisor rather than of their own progression. Here the authors divided: some considered a formal entrustment scale the obvious remedy, while others judged that at current caseloads it would generate documentation without changing the underlying judgment, and that trainer calibration—supervisors jointly assessing the same recorded procedure—would achieve more at lower cost. This disagreement is recorded rather than resolved.

Simulation access. Basic pelvic-trainer access is available at every center represented, and all ten authors regard simulation as established practice for basic psychomotor and suturing skills, consistent with the evidence summarized in Section 4.3. Access arrangements, however, differ materially. At one center a dedicated skills laboratory is open to trainees outside scheduled sessions; at the others, simulator use is organized around periodic courses, so that practice is concentrated rather than distributed. Procedure-specific and virtual-reality models were reported as available only intermittently and largely through externally funded courses. Two obstacles were named repeatedly and by authors in both countries: the absence of supervised simulator time within working hours, which pushes practice into the trainee’s own time, and the absence of any requirement to record simulator performance, which means simulation contributes nothing to the assessment record even where it is used well.

Feedback culture. No center represented has a written policy mandating a specific feedback model. Pendleton’s rules are familiar to most of the authors, but their use reflects individual trainer initiative rather than institutional requirement, and several authors noted that feedback in practice compresses to a brief comment at the end of a case. The obstacle named most often, in both countries, was protected time: structured feedback competes directly with a full operating list, and a model that requires the trainee to speak first is the part most readily dropped under time pressure. The authors again divided on the remedy. Some argued that a mandated model, however imperfectly applied, would at least establish a floor; others argued that mandating it without protecting the time for it produces a documented conversation rather than a useful one. What all agreed on is that educational competence, as argued in Section 4.2, is a distinct and assessable skill rather than an automatic by-product of clinical seniority, and that whether a center’s feedback culture functions depends heavily on whether its senior trainers received any structured training in education themselves.

External review and certification. Both countries operate under EBCOG’s European Training Requirements, and the authors nonetheless report substantial differences in how external review and formal certification bear on daily practice. EBCOG Hospital Visitation had not been undertaken at any of the centers represented during the period the authors describe, and GESEA certification is pursued by individual trainees on their own initiative and at their own expense rather than being embedded in the residency curriculum or required for its completion. The authors converged on the observation that certification therefore functions as a marker of individual motivation rather than as a program quality indicator, which is precisely the opposite of its intended role. They diverged on what should follow. Some hold that external review and GESEA-type certification should become mandatory across the EU, on the grounds that voluntary schemes are adopted first by the centers that need them least; others hold that mandating review without funding the capacity to perform it, and without protecting trainer time to prepare for it, would produce formal compliance without substantive change, and would consume goodwill that QA in these systems cannot easily replace. This divergence, observed directly rather than derived from the literature, illustrates concretely the harmonization gap discussed in Section 9.1: even within a single European regulatory framework, day-to-day QA practice is not standardized, and practitioners inside that framework do not agree on how it should be closed.

The principal findings are presented in Table 1.

Taken together, the authors’ collective opinion is that the conceptual architecture of QA reviewed in Section 3 and Section 4 is sound and broadly fit for purpose, and that its translation into consistent daily practice depends on local leadership, protected time, and institutional priority rather than on the existence of the frameworks themselves. In the authors’ shared view this is the most actionable near-term lesson for training providers, ahead of the more structural harmonization discussed in Section 9. Two qualifications should be carried with it. First, none of the above is measured: these are the reported perceptions of ten trainers, and a center that recognizes itself in these descriptions may differ materially from one that does not. Second, the authors are describing their own institutions, which introduces an obvious risk of favorable reporting; the points of divergence recorded above are, if anything, the more reliable part of the synthesis, because they were arrived at against the group’s evident preference for a common position.

## 5. Monitoring, Evaluation, and Audit of Training Quality

Monitoring, evaluation, and audit are core regulatory functions tied to QA and, like training itself, are properly understood as processes rather than one-off events. Donald Kirkpatrick’s four-level model remains one of the most widely used frameworks for training evaluation: it assesses learners’ immediate reaction (the relevance of aims, the facilitator’s approachability and delivery, and the perceived value and transferability of learning to the workplace); learning results (the knowledge, skills, and attitudes acquired, set against the initial goals); behavior in the workplace (the transferability of those results into actual practice); and business results (the ultimate impact on healthcare delivery) [19,32].

Effective quality assurance depends on the use of measurable indicators that allow continuous monitoring of educational performance and program effectiveness. These indicators should evaluate multiple dimensions of training, including learner satisfaction, knowledge acquisition, technical competency, faculty performance, and patient-related outcomes. Examples of quality indicators applicable to gynecological laparoscopic training programs are presented in Table 4.

In practice, an approved training program is typically reviewed periodically, often within a five-year cycle, by a team of expert, experienced reviewers with no prior connection to the provider. Evidence is assembled, site visits conducted, and minutes prepared; if the review is satisfactory, approval is renewed as part of the mandatory periodic cycle, and the whole process is summarized in a periodic, usually annual, quality assurance report. Where certain standards or indicators are found not to be met, or where good practice could be enhanced or extended, an action plan becomes mandatory: after appropriate dialogue with the provider, remedial actions with defined timescales are agreed, ideally collaboratively, and a follow-up monitoring process verifies compliance. A well-constructed action plan is concise, highlights existing good practice, and specifies measurable, time-limited, and review-scheduled outcomes, with improvement of training and its results as the aim [14,19].

Published evidence connecting these indicators to measurable change is, however, considerably thinner than the number of available indicators suggests, and it is worth being precise about what has and has not been shown. At the level of individual skill acquisition, the evidence is reasonably strong. A randomized controlled trial of a standardized laparoscopy curriculum for gynecology residents demonstrated improvement in technical performance in the operating room relative to conventional training, and a systematic review and meta-analysis of simulation-based gynecologic surgical training found transfer of simulator-acquired skill to intraoperative performance [1,18]. Validated instruments exist for this population: OSATS-type global rating and task-specific scales, the GESEA used for certification, and the Essentials in Minimally Invasive Gynecology manual skills examination, for which pilot validation data have been published [15]. Video-based training in error-recognition and coping strategies has likewise been evaluated in a randomized design with performance endpoints [27]. A program wishing to demonstrate that its trainees are acquiring skill therefore has instruments to do so.

At the level of the training system, the evidence is weak, and this distinction is the one most often blurred. We did not identify a study that takes the implementation of a QA framework as the exposure and measures trainee certification rates, operative volume as first surgeon, procedure-related complication rates, or trainee attrition as the outcome. Survey work has established that trainees and consultants in the UK do not agree on which laparoscopic skills should be required at completion of training, which indicates that even the endpoint against which such a study would be judged is not settled [33]. A practical consequence follows for programs wishing to evaluate their own QA activity. The realistic near-term design is a before-and-after evaluation within a single program, using GESEA or OSATS scores, entrustment level for defined activities, logbook-verified operative volume, and the departmental complication audit as parallel endpoints, with the explicit recognition that such a design cannot separate the effect of the QA intervention from secular changes in trainee cohort, case mix, or technology. Multicenter, prospective, outcome-linked evaluation remains the design the field needs and does not yet have; this is developed in Section 9.1.

At the level of the individual trainee, postgraduate competencies expected by completion of training include the ability to diagnose, manage delivery, and treat patients in the delivery room and admission ward, to discuss the available treatment options, and to determine the indication for, and performance of, specific skills; under the EBCOG curriculum, the trainee is expected to perform 30 laparoscopies as first surgeon during the training period [3].

## 6. Documentation, Certification, Financing, and Governance

Documentation underpins every step described above and can itself be a source of quality standards. The European Academy of Gynecological Surgery and the European Society for Gynecological Endoscopy (ESGE) jointly developed a two-level diploma for trainees completing the laparoscopic curriculum—the Gynecological Endoscopic Surgical Education and Assessment (GESEA) program, with Level 1 aimed specifically at trainees (and suitable for all endoscopists) and Level 2 open to both gynecologists and reproductive surgeons [3]. More broadly, every new postgraduate trainee in Europe arguably needs documentary recognition confirming that they have been adequately trained and are able to practice laparoscopic gynecological surgery safely and independently [3].

Financing underlies the quality management of training and is itself within the scope of QA: relevant standards address funding sources, allocation, and financial management for the institution, for trainers and trainees individually, and for the wider societal impact of training investment [14].

Governance of training QA begins at the institutional level and extends to the national level, where a dedicated working group organized by national authorities is preferable [3]. Relevant tasks at this level include issuing guidelines and regulations, providing local-level assistance, and linking with international bodies for comparison and collaboration. At a still broader, European scale, EBCOG plays a governance role through Hospital Visitation programs intended to quality-assure training [34]. This function currently operates on a voluntary basis; it is reasonable to anticipate that, in time, it could become a mandatory requirement across the EU for all training units to undergo regular quality assurance review.

## 7. Building a Sustainable Culture of Quality

The standards reviewed above are, in the end, instruments in service of a simple goal: quality in adult learning cannot be achieved at its highest level without the active involvement of everyone in the process. Shaping training to a learner’s actual starting point satisfies the learner in the short term, but it is the proposal of higher, attainable targets that sustains the inner motivation needed for continued improvement [35]. An appropriately designed learning environment provides the support and safety that allow this motivation to translate into development; well-chosen, fair educational opportunities, combined with rehearsal and deliberate practice, increase both the pleasure of learning and the trainee’s sense of growing responsibility for their own progress [36]. A genuine culture of feedback and of acknowledgement engages the trainee and the trainer together and sustains both in their respective roles [35,36].

Crucially, the motivating effect of well-designed training extends beyond the formal boundaries of any single course: trainees and trainers alike often experience a sense of personal fulfillment that outlasts the scheduled program itself [37]. Teamwork, interaction, participation, an appropriately stimulating environment, and healthy competition are, on this account, not incidental features of good training but necessary ingredients for its sustained future development [38].

## 8. Limitations

This article has several limitations, most of them inherent to its design, and we set them out in the order in which they constrain the claims made above.

First, the literature component was identified through targeted and purposive rather than systematic searching. Inclusion and exclusion criteria are stated in Section 2.2 and screening counts are reported, but no formal risk-of-bias or quality appraisal of included sources was performed, no second reviewer independently verified the final inclusion set, and selection bias cannot be excluded. Readers should treat the reference base as a defensible map of the field rather than as a complete one.

Second, the multicenter synthesis presented in Section 4.5 draws on the direct teaching and clinical experience of ten authors across several centers, and was collected and coded by a documented procedure, but it remains a qualitative synthesis of expert reflection rather than a Delphi process or a quantitatively validated consensus method. There was no iterative re-rating, no anonymization between authors, and no statistical measure of agreement. The authors were also describing their own institutions, which introduces a risk of favorable reporting that no feature of the method controls for. These observations are not independently verifiable in the way that empirical outcome data would be and should not be cited as evidence of practice in the countries concerned.

Third, and importantly given the emphasis this article places on trainee-centered training in Section 4.3, the resident’s voice is absent. All ten authors hold trainer, supervisor, or clinical leadership roles; no data were collected from trainees themselves, and no trainee co-authored this article. Everything reported here about how trainees experience entrustment, simulator access, and feedback is therefore inferred from the supervisor’s side of the relationship. Given that the divergences most likely to matter—whether feedback is experienced as useful, whether entrustment is experienced as fair—are precisely those a supervisor is least well placed to judge, this is a substantive limitation and not a formality. Collecting the trainee perspective directly is, in our view, the first correction that any extension of this work should make.

Fourth, several of the general QA concepts used here originate in the literature on health promotion, survey methodology, and emergency-medical-services dispatch rather than in surgical or gynecological training. We flag these as conceptual at the point of use, and we regard the underlying principles as transferable, but the transfer has not been demonstrated empirically in this field.

Fifth, the institutional frameworks discussed—EBCOG, EBCOG-PACT, GMC, ESHRE, ESGE/GESEA—are European or UK-centric, and the authors’ own multicenter experience is confined to Romania and Germany, both of which operate inside the same EU regulatory environment, share the European Working Time Directive, and are subject to the same European Training Requirements. Observations drawn from these two settings cannot be generalized to training centers operating under different regulatory frameworks outside the EU, where the accreditation body, the funding model, the trainee-to-case ratio, and the legal basis of supervision may all differ. Section 9.2 addresses what adaptation would involve, but it does so as reasoned argument, not as evidence from those settings.

Sixth, this article shares an author group and a general subject area with our earlier work on standards of care in gynecologic laparoscopic surgery [26]. The distinction in scope is stated in Section 1: the earlier paper addressed standardization of surgical practice, whereas this one addresses quality assurance of the training system, and adds the structured multicenter synthesis and the outcome-evidence appraisal that the earlier work did not contain. Readers should nonetheless be aware of the overlap in perspective that a shared author group implies.

Finally, and most consequentially for future research, we did not identify empirical studies directly linking the implementation of specific QA standards to measurable improvements in trainee competency or patient outcomes in gynecological laparoscopy. As set out in Section 5, evidence exists at the level of individual skill acquisition but not at the level of the training system. The case for QA in this field accordingly rests on its logical coherence and on its track record in adjacent domains such as gastrointestinal endoscopy and health promotion, rather than on outcome data specific to gynecological laparoscopic training. Every recommendation in this article should be read against that limitation, which leads directly into the following section.

## 9. Future Directions

### 9.1. Harmonization of Quality Assurance Frameworks

Quality assurance offers gynecological laparoscopic training a coherent framework spanning the training provider, the trainer, the training process, and the trainee, supported by established tools for monitoring, evaluation, documentation, and certification [26,33]. The available frameworks—EBCOG’s training requirements, the EBCOG-PACT curriculum, and the Hospital Visitation program; the ESGE/GESEA certification pathway; ASGE’s quality indicators; and the GMC’s standards for education providers and for curriculum design—already supply much of the architecture required [3,9,10,31,34,39]. What remains incomplete is, first, the move from largely voluntary external review toward a more consistently harmonized system across training units and countries and, second, the generation of prospective, outcome-based evidence connecting specific QA interventions—simulator-based curricula, structured feedback models such as Pendleton’s rules, defined logbook checkpoints, or periodic institutional audit—to measurable gains in trainee competency and, ultimately, patient outcomes [33]. The order of those two matters. On the evidence currently available, and as our own author group’s disagreement in Section 4.5 illustrates, the case for making external review mandatory is a reasoned position rather than a demonstrated one, and mandating it ahead of the evidence risks producing formal compliance without substantive improvement. We therefore suggest that future work prioritize multicenter, outcome-linked evaluation of laparoscopic training curricula, designed prospectively and with trainee competency and patient outcomes as explicit endpoints, and that harmonization proceed in step with what those evaluations show rather than ahead of them.

### 9.2. Adapting Quality Assurance to Resource-Limited and Non-European Training Systems

The frameworks discussed in this article were developed in, and for, well-resourced European and UK systems, and they carry embedded assumptions that do not hold everywhere: a dedicated skills laboratory, a trainee-to-trainer ratio low enough to permit progressive supervised entrustment, protected time for trainers, a funded external review body, and a legal framework that permits graded independent practice. Transplanting the framework wholesale into a low- or middle-income setting is therefore likely to fail, and to discredit QA in the process; the wider literature on surgical capacity and on health-professional education reform makes the same point about importing high-income models without adapting them [40,41]. The more useful question is which components transfer, and in what order.

Separating the framework by cost is a practical way to answer it. Several components carry little or no direct cost and could reasonably be adopted first: an explicit written statement of learning outcomes; a logbook with defined intermediate checkpoints rather than a single end-of-training review, which, as our own synthesis suggests, is the variable that most determines whether documentation functions; a named educational supervisor for each trainee; a structured feedback model such as Pendleton’s rules, which requires trainer training but no equipment; and reciprocal peer review of training between two departments in the same region, which substitutes mutuality for a funded inspectorate. Other components are capital-intensive—procedure-specific simulation models, virtual-reality platforms, and animal-model training—and are reasonably deferred. Box-trainer simulation occupies a middle position: low-cost pelvic trainers, including locally manufactured ones, support basic psychomotor and suturing skill acquisition, and the evidence for transfer of basic skills does not depend on high-fidelity equipment [18,28].

Three implementation challenges recur across settings and deserve to be named explicitly rather than left to the discussion of resources. The first is trainer time: QA activity added to an unchanged clinical workload is absorbed as unpaid trainer effort and is not sustainable, so protected time is a precondition rather than a refinement, and this constraint binds in well-resourced systems too. The second is the ratio of trainees to laparoscopic cases: where operative volume per trainee is low, competency-based progression cannot function as designed, and a hybrid model—simulation substituting for part of the caseload, with explicit entrustment decisions replacing case-number thresholds—is more realistic than either a purely time-based or a purely volume-based alternative. The third is external review capacity: where no national body exists, regional peer-review consortia and remote review of anonymized logbooks and assessment records offer a lower-cost entry point, and digital platforms make this materially easier than it was a decade ago. We would suggest that any attempt to extend harmonized QA beyond the European context begin by identifying which of these three constraints binds in the setting concerned, and adapt the standards to it, rather than beginning with the standards document and treating local conditions as deviations from it.

### 9.3. Digitalization and Artificial Intelligence in Quality Assurance

This subsection is prospective. Unlike Section 3, Section 4, Section 5 and Section 6, it is not grounded in standards currently in force, and none of the developments discussed below has been evaluated as a quality assurance intervention in gynecological laparoscopic training, and the promise and the perils of these tools in surgery have so far been argued more often than tested [42]. They are set out as hypotheses about the direction the field may take and should be read as such rather than as a logical continuation of the standards presented earlier.

Recent scoping reviews mapping artificial intelligence (AI) applications in minimally invasive surgery training provide a contemporary picture of what has been built and tested [43,44]. Work to date clusters around automated assessment of technical performance—analysis of instrument motion, procedural phase recognition, error detection, and operative efficiency—and around the generation of feedback from that analysis [43]. Proof-of-concept work in laparoscopic surgery has shown that a deep-learning model can assess an intraoperative safety criterion, the critical view of safety in cholecystectomy, with reasonable agreement against expert raters [45]. Reviews of the assessment literature more broadly find that most systems remain at the stage of technical validation on curated datasets, with limited prospective evaluation inside live training programs and little evidence on whether AI-generated feedback changes trainee behavior or downstream operative performance [44]. In parallel, virtual-reality environments and machine-learning-assisted feedback may in principle support continuous competency monitoring and individualized learning pathways, and automated frameworks may allow trainee progression to be tracked across simulation laboratories, structured skills courses, and supervised operating room activity [43,46].

The plausible QA benefit is a reduction in inter-observer variability and the generation of reproducible performance metrics that complement, rather than replace, trainer judgment. The plausible risks are equally identifiable and less often discussed, and a QA framework that adopts these tools should address them explicitly. Models trained on data from high-volume centers may not transfer to different equipment, case mix, or patient populations. Automated metrics measure what is easy to measure, and a program that optimizes toward them may drift away from the competencies that matter, including the non-technical ones. Continuous capture of operative performance raises questions of trainee consent, data ownership, retention, and the permissible use of training data in appraisal or employment decisions that current educational governance frameworks do not answer. Digital credentialing, electronic portfolios, cryptographically secured credentials, and continuously updated competency records may allow institutions, societies, and regulators to verify qualifications more efficiently and to support lifelong learning; we note that this remains an expectation rather than a demonstrated finding [44].

Given the above, the defensible position for a training program today is not to wait for mature AI-based assessment, nor to adopt it because it is available, but to put in place the conditions that would make later adoption both possible and safe: structured and retrievable assessment records, consistent use of validated scoring instruments so that a comparison baseline exists, and explicit governance of who may access trainee performance data and for what purpose. These are conventional QA measures with immediate value independent of whether the technology matures as anticipated.

## 10. Conclusions

Beyond individual standards, assessment tools, and accreditation systems, quality assurance in gynecological laparoscopic training is best understood as a culture embedded across the whole educational pathway. Effective programs depend on the coordinated interaction of institutions, trainers, trainees, governance structures, and continuous evaluation mechanisms, working toward a common objective: the development of competent surgeons capable of delivering safe, high-quality care to women undergoing minimally invasive gynecologic procedures.

Two qualifications should be carried forward from this article. First, the case for QA in this field currently rests on conceptual coherence and on evidence from adjacent domains rather than on outcome data specific to gynecological laparoscopy: we identified no study linking the implementation of a specific QA standard to measurable gains in trainee competency or patient outcomes. Proposals for harmonized or mandatory external review should therefore be presented, and read, as reasoned positions awaiting prospective evaluation, not as evidence-based recommendations. Second, the observations we contribute from our own centers are the reflections of trainers and supervisors in two European countries operating inside a single regulatory framework; they describe how published standards meet daily practice in those settings and are not a generalizable account, least of all for systems outside the EU.

What follows from both qualifications is a research agenda rather than a recommendation: prospective, multicenter, outcome-linked evaluation of laparoscopic training curricula, with trainee competency and patient outcomes as explicit endpoints, and with the trainee’s own perspective collected directly rather than inferred from the supervisors. Until such evidence exists, the strongest claim our own experience supports is a modest one: the frameworks are sound, and the limiting factor is whether institutions choose, and are resourced, to apply them. We would add that those frameworks are themselves moving: the European Training Requirements for obstetrics and gynecology were revised in April 2026, and any program setting up a quality assurance cycle should work from that current edition rather than from the version in force when this article was drafted [47].

## Figures and Tables

**Table 1:** Points of convergence and divergence in the authors’ structured reflections on quality assurance practice (*n* = 10 respondents; five themes derived by inductive coding, Section 2.3).

Theme	Points of Convergence Across Centers	Points of Divergence or Uncertainty
Documentation vs. enforcement	Written requirements (trainee-to-trainer ratio, logbook content, expectation of structured feedback) exist everywhere and are more complete than their routine enforcement; no center has a written escalation procedure for unmet requirements.	Interval of verification: review tied to a recurring annual appraisal in the German center versus predominantly end-of-training review in the Romanian centers.
Entrustment and supervision	Entrustment decisions rest on the individual supervisor’s observation rather than on a written program-level scale; rotating trainees experience a visible difference in the pace of entrustment.	Whether a formal entrustment (EPA) framework is practicable at current caseloads, or whether trainer calibration would achieve more at lower cost; the authors were divided.
Simulation access	Basic pelvic-trainer access is universal, and simulation is regarded as established for psychomotor and suturing skills; simulator performance is not recorded in the assessment record at any center.	Whether simulator time is scheduled and supervised or left to trainee initiative, and the availability of procedure-specific and virtual-reality models.
Feedback culture	No center mandates a specific feedback model; structured feedback reflects individual trainer initiative, and lack of protected time was the obstacle named most often in both countries.	Whether mandating a model would establish a useful floor or generate a documented conversation without protected time to conduct it; the authors held opposing views.
External review and certification	EBCOG Hospital Visitation had not been undertaken at any represented center in the period described, and GESEA certification is pursued individually rather than embedded in the curriculum, so it marks trainee motivation rather than program quality.	Whether external review and GESEA-type certification should become mandatory EU-wide; concerns were raised about funding, review capacity, and formal compliance without substantive change.

**Table 2:** Core dimensions of quality assurance and their application to gynecological laparoscopic training.

QA Domain	General Healthcare Objective	Application in Laparoscopic Training	Example Indicator
Structure	Adequate resources and infrastructure	Simulation laboratories, laparoscopic equipment, trained faculty	Number of simulators per trainee
Process	Standardized educational delivery	Curriculum implementation, supervised procedures, structured feedback	Percentage of completed training modules
Outcome	Achievement of predefined goals	Competency acquisition and patient safety	Certification success rate
Monitoring	Continuous quality control	Regular audits and trainee evaluations	Annual QA reports
Improvement	Identification of deficiencies	Corrective actions and curriculum updates	Number of implemented action plans

**Table 3:** Quality assurance standards according to stakeholder responsibilities in gynecological laparoscopic training.

Stakeholder	Main Responsibilities	Quality Standards
Training Institution	Curriculum delivery, infrastructure, governance	Accredited program, adequate facilities, simulator access, trainee support
Trainer	Teaching and supervision	Clinical competence, educational competence, CPD participation, structured feedback
Trainee	Active participation in learning	Attendance, competency progression, logbook completion, professionalism
Accrediting Bodies	External oversight	Program evaluation, certification, periodic review
Healthcare System	Governance and financing	Resource allocation, regulatory compliance, workforce planning

**Table 4:** Outcome-oriented quality indicators for competency-based gynecological laparoscopic training programs.

Domain	Indicator	Measurement Method
Trainee Satisfaction	Course evaluation score	Standardized questionnaires
Knowledge Acquisition	Written examination performance	MCQ examinations, oral assessment
Technical Skills	Simulation assessment score	GESEA and OSATS evaluation
Clinical Performance	Number of supervised procedures completed	Logbook review
Competency Progression	Entrustable Professional Activities (EPA) achievement rate	Workplace-based assessment
Competency Achievement	Certification success rate	Examination outcomes
Professional Development	Portfolio completion and reflective practice	Competency portfolio review
Faculty Performance	Trainer evaluation score	Anonymous trainee feedback
Patient Safety	Procedure-related complication rate	Clinical audit
Program Quality	Accreditation status and QA compliance	External review and site visits
Continuous Improvement	Implementation of corrective actions	Annual QA reports and action-plan monitoring

## Data Availability

The original contributions presented in this study are included in the article. Further inquiries can be directed to the corresponding author.

## Abbreviations

The following abbreviations are used in this manuscript:

QA	Quality assurance
EBCOG	European Board and College of Obstetrics and Gynecology
UEMS	Union Européenne des Médecins Spécialistes (European Union of Medical Specialists)
ESGE	European Society for Gynecological Endoscopy
ASGE	American Society for Gastrointestinal Endoscopy
GESEA	Gynecological Endoscopic Surgical Education and Assessment
ESHRE	European Society for Human Reproduction and Embryology
GMC	General Medical Council
GRS	Global Rating Scale
ISO	International Organization for Standardization
CBME	Competency-based medical education
EPA	Entrustable Professional Activity
CPD	Continuing professional development
OSATS	Objective Structured Assessment of Technical Skills
MCQ	Multiple-choice question
CCT	Certificate of Completion of Training
AI	Artificial intelligence

## References

[1] Orejuela F.J., Aschkenazi S.O., Howard D.L., Jeppson P.C., Balgobin S., Walter A.J., White A., Olivera C.K., Sanses T.V., Thompson J. (2022). Gynecologic surgical skill acquisition through simulation with outcomes at the time of surgery: A systematic review and meta-analysis. Am. J. Obstet. Gynecol..

[2] de Jonge V., Kuipers E.J., van Leerdam M.E. (2012). Quality assurance in the endoscopy unit: The view of endoscopy personnel. Frontline Gastroenterol..

[3] Standing Committee on Training and Assessment (2018). European Training Requirements in Obstetrics and Gynaecology (UEMS Document 2018/18).

[4] Li X., Li G., Yang C. (2026). Effectiveness and implementation of simulation training in obstetric and gynecological surgery education: Systematic review and meta-analysis. Front. Med..

[5] Edmonds K., Warner S., Endicott S. (2024). Advances in gynecologic simulation: Implementation, validity, and new resources. Curr. Opin. Obstet. Gynecol..

[6] Aaberg C., Dahmen H., Davies C., Sandau P.L., Srinivasan R. (2021). ISO 9001:2015 versus ICH Q10-A comparison. PDA J. Pharm. Sci. Technol..

[7] Üstün T.B., Chatterji S., Mechbal A., Murray C.J.L. (2002). Quality assurance in surveys: Standards, guidelines and procedures. Household Sample Surveys in Developing and Transition Countries.

[8] Ferreira D.C., Vieira I., Pedro M.I., Caldas P., Varela M. (2023). Patient Satisfaction with Healthcare Services and the Techniques Used for its Assessment: A Systematic Literature Review and a Bibliometric Analysis. Healthcare.

[9] Day L.W., Cohen J., Greenwald D., Petersen B.T., Schlossberg N.S., Vicari J.J., Calderwood A.H., Chapman F.J., Cohen L.B., ASGE Endoscopy Unit Quality Indicator Taskforce (2017). Quality indicators for gastrointestinal endoscopy units. VideoGIE.

[10] Van der Aa J.E., Goverde A.J., Scheele F. (2018). Improving the training of the future gynaecologist: Development of a European curriculum in Obstetrics and Gynaecology (EBCOG-PACT). Facts Views Vis. Obgyn.

[11] Gnanalingham J., Gnanalingham M.G., Gnanalingham K.K. (2001). An audit of audits: Are we completing the cycle?. J. R. Soc. Med..

[12] Catford J. (1993). Auditing health promotion: What are the vital signs of quality?. Health Promot. Int..

[13] Speller V., Evans D., Head M. (1997). Developing quality assurance standards for health promotion practice in the UK. Health Promot. Int..

[14] Endalamaw A., Khatri R.B., Mengistu T.S., Erku D., Wolka E., Zewdie A., Assefa Y. (2024). A Scoping Review of Continuous Quality Improvement in Healthcare System: Conceptualization, Models and Tools, Barriers and Facilitators, and Impact. BMC Health Serv. Res..

[15] Munro M.G., Brown A.N., Saadat S., Gomez N., Howard D., Kahn B., Stockwell E., Advincula A.P., Volker W., Thayn K. (2020). Essentials in Minimally Invasive Gynecology Manual Skills Pilot Validation Trial. J. Minim. Invasive Gynecol..

[16] Hilsden R.J., Rostom A., Dubé C., Pontifex D., McGregor S.E., Bridges R.J. (2011). Development and implementation of a comprehensive quality assurance program at a community endoscopy facility. Can. J. Gastroenterol..

[17] Green L.W., Brooks-Bertram P. (1978). Peer review and quality control in health education. Health Values Achiev. High Level Wellness.

[18] Shore E.M., Grantcharov T.P., Husslein H., Shirreff L., Dedy N.J., McDermott C.D., Lefebvre G.G. (2016). Validating a standardized laparoscopy curriculum for gynecology residents: A randomized controlled trial. Am. J. Obstet. Gynecol..

[19] World Federation for Medical Education (2023). WFME Global Standards for Quality Improvement: Postgraduate Medical Education, 2023 Revision.

[20] Argentino G.L.S., Bueloni-Dias F.N., Leite N.J., Peres G.F., Elias L.V., Bortolani V.C., Padovani C.R., Spadoto-Dias D., Dias R. (2019). The role of laparoscopy in the propaedeutics of gynecological diagnosis. Acta Cir. Bras..

[21] Al-Omary H., Soltani A., Stewart D., Nazar Z. (2024). Implementing learning into practice from continuous professional development activities: A scoping review of health professionals’ views and experiences. BMC Med. Educ..

[22] Ten Cate O. (2017). Competency-Based Postgraduate Medical Education: Past, Present and Future. GMS J. Med. Educ..

[23] Caccia N., Nakajima A., Scheele F., Kent N. (2015). Competency-Based Medical Education: Developing a Framework for Obstetrics and Gynaecology. J. Obstet. Gynaecol. Can..

[24] Gomez-Garibello C., Wagner M., Seymour N., Okrainec A., Vassiliou M. (2023). The Entrustable Professional Activities of Laparoscopic Surgery: Moving toward an Integrated Training Model. Surg. Endosc..

[25] Shah N., Desai C., Jorwekar G., Badyal D., Singh T. (2016). Competency-Based Medical Education: An Overview and Application in Pharmacology. Indian J. Pharmacol..

[26] Tica V.I., Tica A.A., De Wilde R.L. (2022). The future in standards of care for gynecologic laparoscopic surgery to improve training and education. J. Clin. Med..

[27] Lang F., Gerhäuser A.S., Wild C., Wennberg E., Schmidt M.W., Wagner M., Müller-Stich B.P., Nickel F. (2023). Video-based learning of coping strategies for common errors improves laparoscopy training: A randomized study. Surg. Endosc..

[28] Goodman A.J., Melson J., Aslanian H.R., Bhutani M.S., Krishnan K., Lichtenstein D.R., Navaneethan U., Pannala R., Parsi M.A., Schulman A.R. (2019). Endoscopic simulators. Technology Status Evaluation Report. Gastrointest. Endosc..

[29] Hart R., Karthigasu K., Garry R. (2007). Virtual reality simulation training can improve technical skills during laparoscopic salpingectomy for ectopic pregnancy. BJOG.

[30] Martire F.G., Ianes I., Costantini E., Zupi E., Lazzeri L. (2026). Abdominal Wall Elevation for Pneumoperitoneum in Laparoscopic Gynecologic Surgery: A Retrospective Comparative Study of Experienced and Trainee Surgeons. J. Minim. Invasive Gynecol..

[31] General Medical Council (2015). Promoting Excellence: Standards for Medical Education and Training.

[32] Ullah H., Huma S., Yasin G., Ashraf M., Tahir-ud-Din Q., Shabana H., Sarfraz J. (2024). Curriculum and program evaluation in medical education: A short systematic literature review. Ann. Med. Surg..

[33] Khan Z.N., Shrestha D., Shugaba A., Lambert J.E., Haslett E., Afors K., Bampouras T.M., Subar D., Gaffney C., Clark T.J. (2025). What laparoscopic skills are necessary for the certificate of completion of training? A prospective nationwide cross-sectional survey of obstetrics and gynaecology and general surgery trainees and consultants in the UK. BMJ Open.

[34] Wladimiroff J.W. (2003). EBCOG hospital visiting, a step forward in the quality assessment of training in obstetrics and gynaecology. Eur. J. Obstet. Gynecol. Reprod. Biol..

[35] Hardie P., O’Donovan R., Jarvis S., Redmond C. (2022). Key Tips to Providing a Psychologically Safe Learning Environment in the Clinical Setting. BMC Med. Educ..

[36] Mash B., Edwards J. (2020). Creating a Learning Environment in Your Practice or Facility. S. Afr. Fam. Pract..

[37] Geerts J.M. (2024). Maximizing the Impact and ROI of Leadership Development: A Theory- and Evidence-Informed Framework. Behav. Sci..

[38] Zamiri M., Esmaeili A. (2024). Strategies, Methods, and Supports for Developing Skills within Learning Communities: A Systematic Review of the Literature. Adm. Sci..

[39] General Medical Council (2017). Excellence by Design: Standards for Postgraduate Curricula.

[40] Frenk J., Chen L., Bhutta Z.A., Cohen J., Crisp N., Evans T., Fineberg H., Garcia P., Ke Y., Kelley P. (2010). Health professionals for a new century: Transforming education to strengthen health systems in an interdependent world. Lancet.

[41] Meara J.G., Leather A.J.M., Hagander L., Alkire B.C., Alonso N., Ameh E.A., Bickler S.W., Conteh L., Dare A.J., Davies J. (2015). Global Surgery 2030: Evidence and solutions for achieving health, welfare, and economic development. Lancet.

[42] Hashimoto D.A., Rosman G., Rus D., Meireles O.R. (2018). Artificial intelligence in surgery: Promises and perils. Ann. Surg..

[43] Caballero D., Sánchez-Margallo J.A., Pérez-Salazar M.J., Sánchez-Margallo F.M. (2025). Applications of Artificial Intelligence in Minimally Invasive Surgery Training: A Scoping Review. Surgeries.

[44] Escobar-Castillejos D., Barrera-Animas A.Y., Noguez J., Magana A.J., Benes B. (2025). Transforming Surgical Training with AI Techniques for Training, Assessment, and Evaluation: Scoping Review. J. Med. Internet Res..

[45] Mascagni P., Vardazaryan A., Alapatt D., Urade T., Emre T., Fiorillo C., Pessaux P., Mutter D., Marescaux J., Costamagna G. (2022). Artificial intelligence for surgical safety: Automatic assessment of the critical view of safety in laparoscopic cholecystectomy using deep learning. Ann. Surg..

[46] Ellaway R., Topps D., Lee S., Armson H. (2015). Virtual patient activity patterns for clinical learning. Clin. Teach..

[47] Standing Committee on Training and Assessment (2026). European Training Requirements for the Specialty of Obstetrics and Gynaecology (UEMS Document 2026/11).

